# Bibliometric evaluation of publications on inflammasomes in atherosclerosis from 2002 to 2022

**DOI:** 10.3389/fcvm.2023.1067226

**Published:** 2023-04-12

**Authors:** Yu Tan, Yanqiao Yu, Wei Liu, Xiaojuan Ma, Dazhuo Shi

**Affiliations:** ^1^ National Clinical Research Center for Chinese Medicine Cardiology, Xiyuan Hospital, China Academy of Chinese Medical Sciences, Beijing, China; ^2^ Department of Cardiology, Beijing Jishuitan Hospital, The Fourth Clinical Medical College of Peking University, Beijing, China

**Keywords:** atherosclerosis, inflammasome, citespace, VOSviewer, bibliometric

## Abstract

**Background:**

Inflammasomes have emerged as an important and promising area of investigation in atherosclerosis. This field, however, lacks bibliometric studies. To help understand how basic and clinical research on inflammasomes in atherosclerosis will develop in the future, we used bibliometric analysis to visualize hotspots and trends.

**Methods:**

Studies related to inflammasomes in atherosclerosis were collected from the Web of Science Core Collection database. Each study was analyzed bibliometrically and visually. CiteSpace and VOSviewer software were used to generate knowledge maps.

**Results:**

A total of 894 articles were identified. Sixty-two countries and 338 institutions led by China and the United States contributed to these publications. The leading research institutions were Harvard Medical School and Columbia University. *Circulation* was the most frequently cited journal in this field. Among the 475 authors determined, Eicke Latz authored the most studies, and Peter Duewell has been cocited the most. NLRP3 inflammasome, NF-kappa B, macrophage and oxidative stress are the most commonly used keywords.

**Conclusion:**

There has been a blooming of research on inflammasomes in atherosclerosis during the last two decades. Future studies will likely explore the molecular mechanism of inflammasomes in cell death. More compellingly, researchers may further delve into the potential clinical value of affecting pathological changes in atherosclerosis by modulating the initial transcription immune response and intracellular multiprotein assembly process of the NLRP3 inflammasome. Our research will be helpful to scholars focusing on inflammation—a much-needed breakthrough in the pathophysiological alterations of atherosclerosis—with a novel perspective.

## Introduction

The inflammasome is an intracellular multimeric complex molecule of approximately 700 kDa that can be activated by cellular infection or stress signals, and it can modify and mature a variety of inflammatory factor precursors, such as pro-IL-1β and pro-IL-18, thereby participating in the human innate immune response and a variety of diseases. Dr Jürg Tschopp and his colleagues first proposed this concept in 2002 (1). Inflammasomes were described for the first time as containing four crucial components, including caspase-1, caspase-5, Pycard, and NALP1 (2). Moreover, it can regulate pyroptosis, which is an inflammatory form of cell death that occurs in the presence of inflammatory or stressful conditions (3). Previous evidence suggests that caspases, proinflammatory proteases, are recruited and activated by the inflammasome when it identifies pathogen-associated molecular patterns (PAMPs) or danger-associated molecular patterns (DAMPs) (4–6). Subsequently, caspase-1 is activated and modifies the predecessors of IL-1β and IL-18 into the corresponding mature cytokines (7, 8). Current findings have identified various types of inflammasomes that play a crucial role in host defenses against multiple pathogens, and in turn, pathogens have evolved numerous mechanisms for inhibiting inflammasome activation (9, 10). In addition, it has been proven that inflammasomes are constructed by pattern recognition receptors (PRRs), which are involved in regulating the immune system (11, 12).

Researchers have extensively studied the biological functions of two types of inflammasomes: canonical inflammasomes, including the NLRP3 inflammasome, NLRC4 inflammasome and AIM2 inflammasome, and noncanonical inflammasomes, which contain caspase-11 and can be triggered by cytosolic LPS (13–15). Currently, three protein bodies have been identified as being part of the inflammasome, including apoptosis-associated speck-like protein containing a CARD (ASC), caspase proteases, and a NOD-like receptor (NLR) family protein (e.g., NLRP3) or HIN200 family protein (e.g., AIM2) (2, 8, 16–18). A typical inflammasome is composed of NLRs as the receptor, ASCs as the adaptor, and caspases as the effector (19). However, receptor protein types are quite diverse. Generally, receptor proteins can be divided into two categories based on their structure: receptor proteins of the NLR family and receptor proteins of the non-NLR family (17, 20–22). PRRs can trigger an inflammatory response when they detect signals (23–25).

The congregation of different inflammasomes can be triggered by different endogenous and exogenous signals. Canonical inflammasomes are stimulated by the corresponding second messengers in the cytoplasm to complete the assembly of protein multimers and eventually activate caspase-1 (26, 27). NLRP3 can be primed by NF-*κ*B signaling, and NLRP3 is assembled and activated after being primed (28). In addition, pathological conditions such as oxidized low-density lipoprotein (oxLDL) and cholesterol can initiate the formation of NLRP3 inflammasomes (11, 18, 29, 30). ASCs may not be necessary for certain NLRC4 functions, but they are required for NAIP-mediated interactions, which are bacterial protein‒ligand receptors, and these interactions determine inflammasome functions (31, 32). AIM2 mediates the immune response against bacterial and viral infection by activating caspase-1 and cleaving IL-1β and IL-18 (33). Several scholars have reported that LPS activates the noncanonical inflammasome in mice, which consists of caspase-11 (15, 28). In addition, it has been demonstrated that caspase-11 induces the NLRP3 inflammasome to cleave pro-IL-1β or pro-IL-18 (14, 33, 34). When caspase-4 is triggered by LPS, inflammasome activation can occur without the need for a second signal (34–36).

The NLRP3 inflammasome has been identified as a key contributor to lipid metabolism disorder and inflammation in cardiovascular disease because it can be triggered by mitochondrial dysfunction, endoplasmic reticulum (ER) stress, ox-LDL and crystalline cholesterol (37, 38). Atherosclerotic plaques are the pathological product of chronic inflammation of the vascular endothelium and lipid metabolism disorder. Essentially, these plaques are made up of foam cells, immune cells, cholesterol crystals, and smooth muscle cells that proliferate under inflammatory conditions (37, 38). Previous research has shown that the relationship between NLRP3 and prognosis in patients with acute coronary syndromes is highly relevant (39). Furthermore, patients undergoing carotid endarterectomy have an increase in NLRP3, ASC, caspase-1, and IL-1β expression in their carotid atherosclerotic plaques (40). The expression of all these molecules was higher in unstable plaques than in stable plaques (41). Zheng et al. suggested that smokers with high cholesterol levels, hypertension, and diabetes overexpress NLRP3 in their aortas (42). In addition to affecting lipid metabolism, the NLRP3 inflammasome also exacerbates inflammation by mediating the upregulation of certain cytokines through the activation of IL-1β and IL-18 (5, 6, 8). For example, IL-1β increases monocyte chemotactic protein-1 and vascular cell adhesion molecule-1-mediated leukocyte-endothelial cell adhesion effects, which in turn enhance monocyte migration across endothelial cells (36, 38). Compared to asymptomatic stable plaques, the number of smooth muscle cells and collagen is lower and the number of lipids and macrophages is higher in unstable plaques (41). This is due to greater expression of inflammasomes in unstable plaques, decreasing smooth muscle cells and collagen and increasing lipids and macrophages (42, 43). Thus, targeting NLRP3 inflammasome signaling might be a novel interventional therapy to attenuate atherosclerosis.

Researchers have been exploring inflammasomes in atherosclerosis for the past 20 years, but some issues remain unrealized. How does the inflammasome participate in the development of atherosclerosis, such as endothelial barrier disruption, inflammatory cell migration across the endothelium, and fibrous cap rupture of unstable plaques? Based on bibliometrics of the WOS Core database, we visualized the research frontiers of inflammasomes related to atherosclerosis in this study. Researchers in the field are discovering the role of the inflammasome in atherosclerosis worldwide, contributing to the formulation of further investigation plans, as well as shedding light on future research.

## Methods

### Search methodologies and data collection

**Figure 1:**
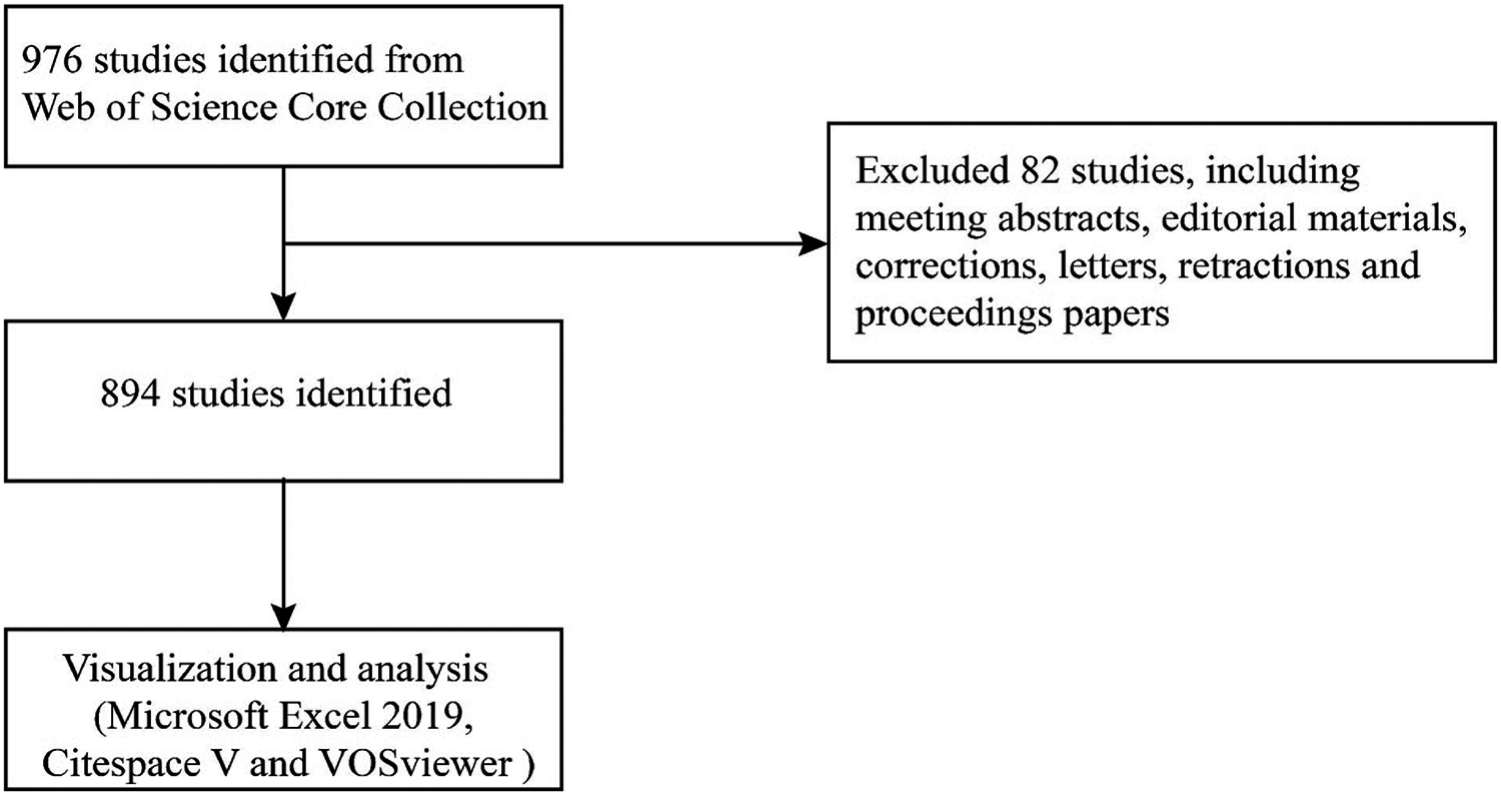
Flowchart of literature selection.

The Science Citation Index Expanded (SCI-EXPANDED) index of the Web of Science Core Collection (WoSCC) was selected to serve as the source database for data retrieval (Table 1) (44). The data were obtained on March 29th from the database mentioned above. In MeSH (https://www.ncbi.nlm.nih.gov/mesh), the term “atherosclerosis” showed other expressions, such as “atherosclerosis” and “arteriosclerosis”. In the WOS database, the following keywords and strategies are used: #1, “inflammasome”; #2, “inflammasomes”; #3 “atherosclerosis”; #4, “arteriosclerosis”; #5 “#1” OR “#2”; #6 “#3” OR “#4”; #7 “#5” AND “#6”. Articles from 2002 to 2022 (March 29th, 2022) were identified. The language setting was English, and the document type was article. Each identified article's data were obtained from the SCI-EXPANDED database, which was then opened in Excel 2016, including authors, affiliations, countries/regions, journals, the number of papers and citations, publication year, keywords, and references. Data were extracted independently by two writers from the qualifying papers (Figure 1).

**Table 1:** Abbreviation list.

Abbreviation	Full name
AS	Atherosclerosis
AF	Atrial fibrillation
CD	Cluster of differentiation
CCs	Cholesterol crystals
DAMPs	Danger-associated molecular patterns
ER	Endoplasmic reticulum
HFD	High-fat diet
HAECs	Human aortic endothelial cells
LDLR	Low-density lipoprotein receptor
NF-*κ*B	Nuclear factor- kappa B
NLRP3	NOD-like receptor family protein 3
oxLDL	Oxidized low-density lipoprotein
PAMPs	Pathogen-associated molecular patterns
ROS	Reactive oxygen species
TLR	Toll-like receptor
TMAO	Trimethylamine-N-oxide
VSMCs	Vascular smooth muscle cells
WoSCC	Web of Science Core Collection

### Bibliometric analysis

Bibliometric and visual analysis was completed by using Microsoft Excel 2019, VOSviewer, and CiteSpace on all valid documents retrieved from WoSCC. Standard competition ranking is used to determine the ranking order. Using VOSViewer and CiteSpace, we performed bibliometric analysis and network visualization. By analyzing keywords and key references published by influential scholars, we have locked the research hotspots and frontiers in this field.

We set CiteSpace's parameters as follows: method (LLR), time slicing (from 2002 to 01-01 to 2022-03-29), years per slice (1), the links are of cosine strength within slices; the pathfinder and the sliced network are pruned, then the merged network is pruned; and sources and nodes can be defined in different ways depending on specific requirements (45). Cooccurrence, clustering, and burst analyses were conducted.

Bibliometric network graphs were constructed and visualized using VOSviewer v1.6.10.0 (Leiden University, Leiden, the Netherlands) (46). For this study, VOSviewer was used to analyze cocited journals. Node sizes indicate publication numbers, line thickness indicates relationship strength, and node colors indicate distinct clusters or eras.

CiteSpace is equipped with cluster analysis, timeline views, countries, references, and keyword citation bursts for visual evaluation of knowledge fields and trend development (47). Cluster analysis can be used to categorize keywords and discover the most relevant topics for studies on inflammasomes in AS. The discovery of new research trends is often based on bursts of countries, references, and keywords.

## Results

### Annual publication

**Figure 2:**
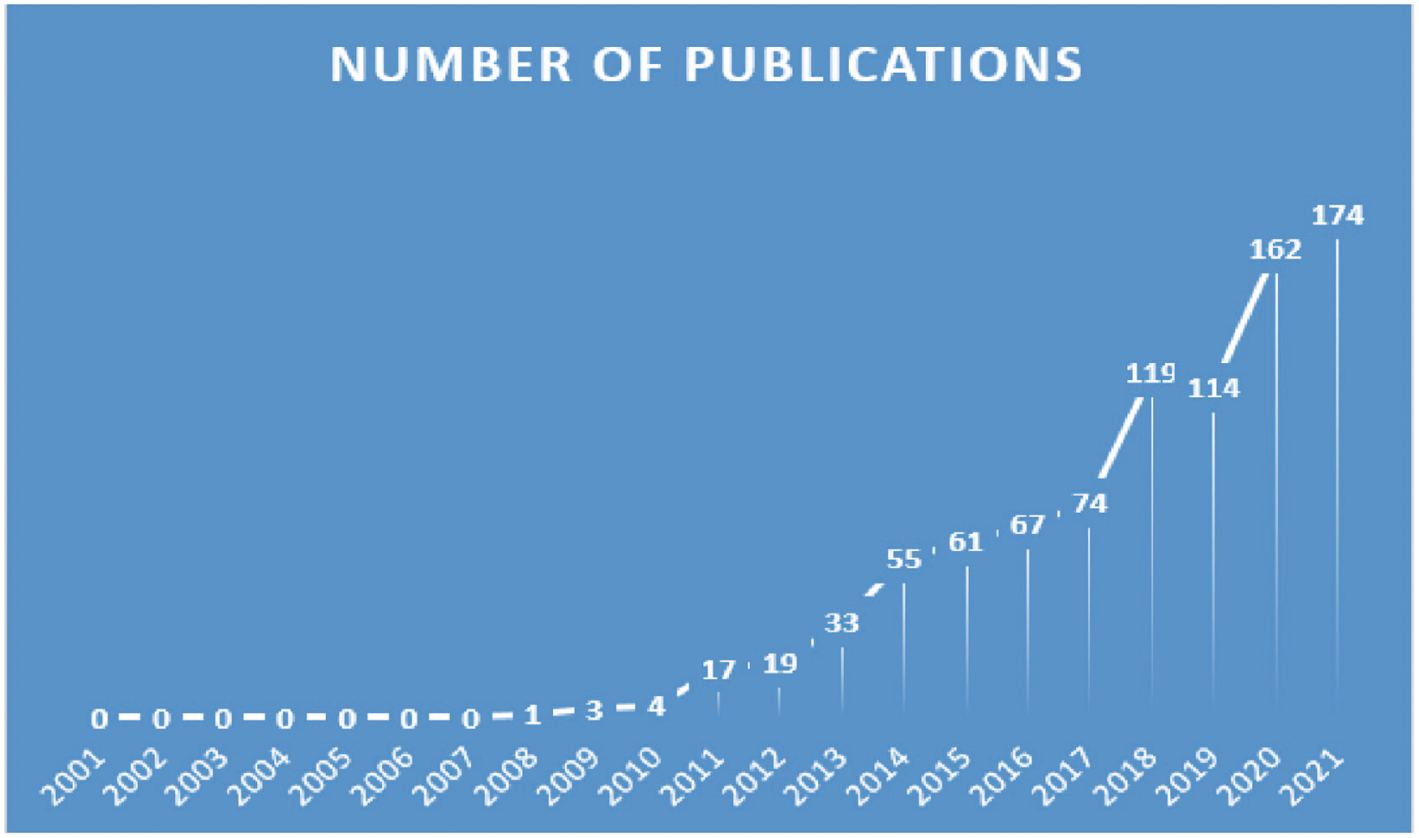
Trends of inflammasomes published in atherosclerosis studies over the past 20 years.

By searching for topic words, 894 related publications were identified. In Figure 2, we show the distribution of publications. During the period of 2007 to 2021, appropriate publications increased annually. Historically, there have been two stages in the development of publications on “inflammasome AND atherosclerosis”. In the first phase, growth occurred slowly and steadily from 2007 to 2018. From 2020 onward, there was a notable increase in publication counts in the second stage from 2019 to 2021. The calculation shows that the frequency of publication more than doubled from 2017 to 2021. While 2022's data are incomplete, the number of articles published in that year is expected to increase rapidly.

### Distribution of countries/regions and institutions

**Figure 3:**
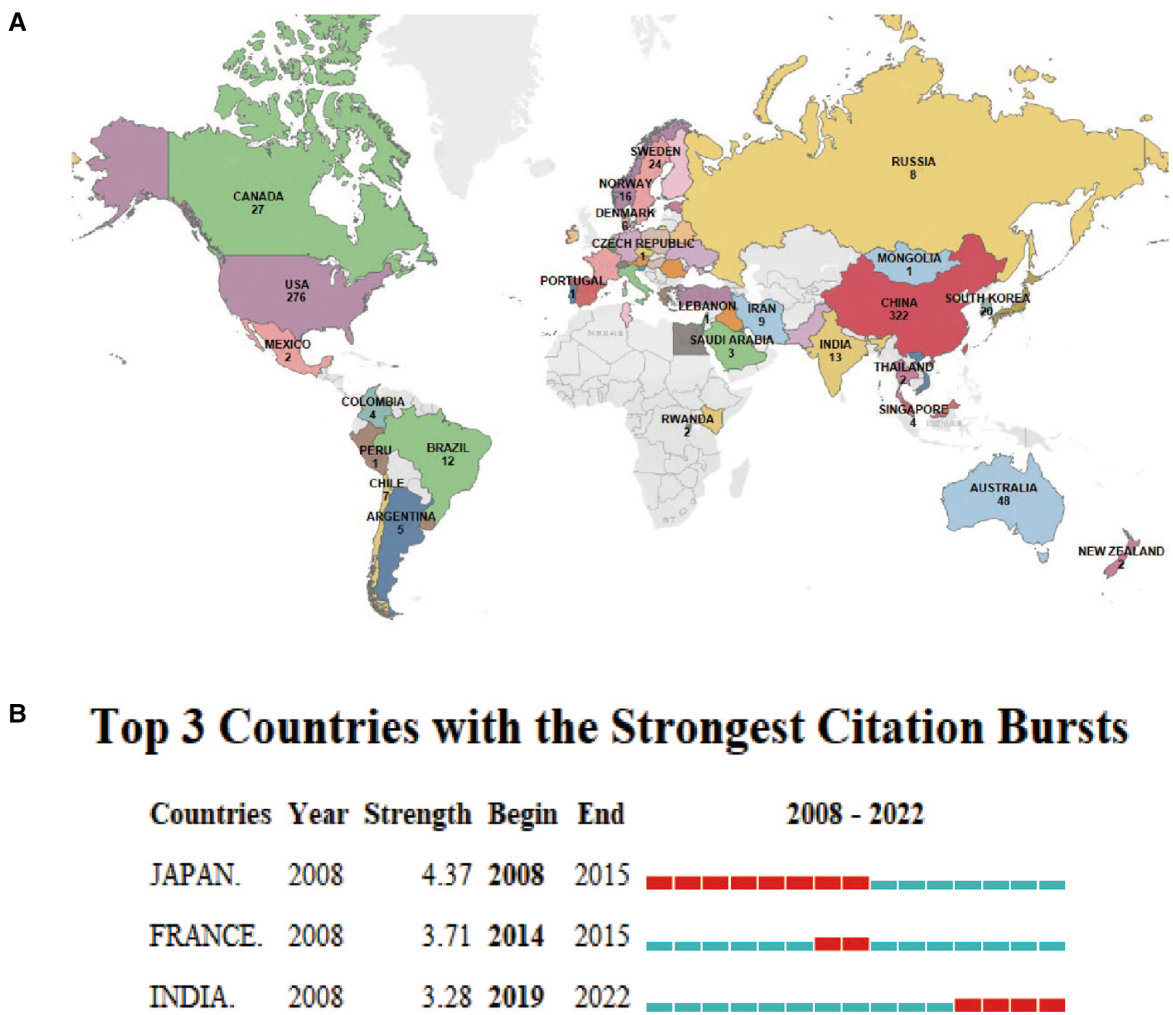
Distribution of global publications in the field of inflammasomes in atherosclerosis. (**A**) The documents of “inflammasome AND atherosclerosis”. The colors indicate the number of documents in each country, while gray represents countries with no publications. (**B**) Top 3 countries/regions with the strongest citation bursts by CiteSpace. ɣ: 1.0, minimum duration: 1. Citation bursts reflect rapid changes in a variable in a short amount of time. Red bars represent the duration of the burst.

The 894 articles were published in 62 countries and 338 institutions (Figure 3A). The statistical analysis indicates that multinational/regional collaboration thrives in this field. The majority of publications come from China (322, 34.40%), as shown in Table 2, and the United States (276, 29.48%), which are at least three times higher than other countries. In addition, Japan showed the highest citation burst (a large change in the number of publications in a short time) strength with 4.37 from 2008 to 2015, indicating that many Japanese scholars emerged to focus on inflammasomes and atherosclerosis. French and Indian scholars had citation bursts of 3.71 and 3.28, respectively; however, India showed a late burst from 2019 to 2022. Until 2022, all three countries/regions experienced strong citation bursts, so many scholars are expected to join the field in the near future.

**Table 2:** Top 10 countries/regions and institutions related to “inflammasome AND atherosclerosis”.

Rank	Country	Year	Centrality	Count (%)	Institution	Year	Centrality	Count (%)
1	China	2012	0.05	322 (34.40%)	Harvard Med Sch (USA)	2017	0.11	22 (2.35%)
2	Usa	2009	0.53	276 (29.48%)	Karolinska Inst (Sweden)	2011	0.04	16 (1.70%)
3	Germany	2010	0.1	86 (9.18%)	Shanghai Jiao Tong Univ (China)	2016	0.02	15 (1.60%)
4	Italy	2013	0.08	55 (5.87%)	Xi An Jiao Tong Univ (China)	2014	0.01	15 (1.60%)
5	Japan	2008	0.05	51 (5.44%)	Univ Bonn (Germany)	2010	0.04	14 (1.49%)
6	Australia	2010	0.07	48 (5.12%)	Columbia Univ (USA)	2014	0.1	13 (1.38%)
7	England	2011	0.07	39 (4.16%)	Univ Cambridge (UK)	2011	0.05	13 (1.38%)
8	Spain	2013	0.02	38 (4.05%)	Dalian Med Univ (China)	2014	0.01	12 (1.28%)
9	Netherlands	2009	0.1	32 (3.41%)	Sun Yat Sen Univ (China)	2012	0.03	12 (1.28%)
10	France	2012	0.04	30 (3.20%)	Univ Massachusetts (USA)	2010	0.02	12 (1.28%)

Table 2 lists the top 10 most productive organizations, four in China, three in the United States, one in Sweden, one in Britain and one in Germany. The most creative institution was Harvard Medical School (22, 2.35%), indicating that the institution has strong leadership in the field of inflammasome research. The data suggest that Harvard Medical School (0.11) and Columbia University (0.1) have strong betweenness centrality, which shows active collaboration with other institutions. Furthermore, there is a high level of cooperation between American institutions, with three of these institutions ranking among the 10 most productive institutions. This has given the United States a great deal of educational influence on this field by leveraging regional advantages to the greatest degree.

### Authors and cocited authors

**Figure 4:**
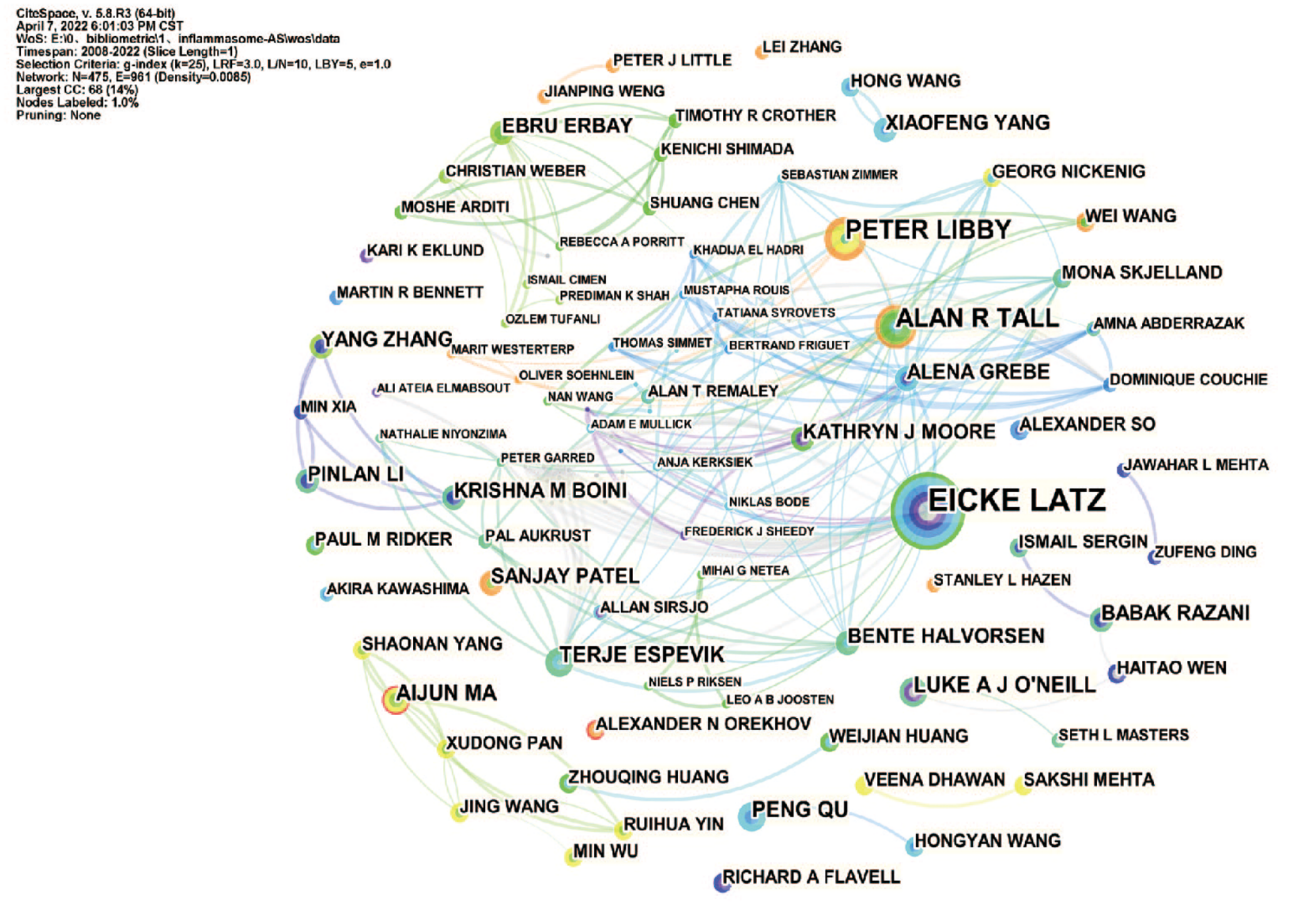
Citespace visualization map of authors involved in “inflammasome AND atherosclerosis”.

In total, 475 authors contributed to the literature on inflammasomes in atherosclerosis. According to Table 3, Eicke Latz published the most papers (16, 1.70%). The centralities of Eicke Latz (0.19) and Peter Libby (0.11) are highest among the top 10 authors, indicating that they have a strong influence on one another's work and other groups' work. Circles represent authors, connections between authors are represented by lines, and colors show the cluster of cooperation between authors. Figure 4 shows a collaborative network between authors, such as Eicke Latz, Terje Espevik, Hajime Kono, George S Abela, Max Schnurr, Cherilyn M Sirois and Gregory Vladimer. Cocited authors are two or more authors that are simultaneously cited in one or more papers; as a consequence, they establish a cocitation relationship. Interestingly, we found 15 researchers with more than 500 citations, which proves that their deep work in this field has been recognized by other scholars (Table 3). Duewell P(450) was the leader, followed by Libby P(267).

**Table 3:** Top 10 authors and cocited authors related to “inflammasome AND atherosclerosis”.

Rank	Author	Count	Centrality	Year	Cocited author	Citation	Centrality
1	EICKE LATZ	16 (1.70%)	0.19	2010	DUEWELL *P*	450	0.01
2	PETER LIBBY	9 (0.96%)	0.11	2017	LIBBY *P*	267	0.00
3	ALAN R TALL	9 (0.96%)	0.02	2015	RIDKER PM	264	0.02
4	AIJUN MA	6 (0.64%)	0.04	2018	MARTINON F	230	0.01
5	LUKE A J O'NEILL	6 (0.64%)	0.05	2012	RAJAMAKI K	185	0.02
6	TERJE ESPEVIK	6 (0.64%)	0.02	2010	ZHOU RB	183	0.01
7	PENG QU	6 (0.64%)	0.01	2015	SCHRODER K	166	0.01
8	KRISHNA M BOINI	5 (0.53%)	0.00	2014	HANSSON GK	152	0.01
9	XIAOFENG YANG	5 (0.53%)	0.00	2015	DINARELLO CA	151	0.01
10	YANG ZHANG	5 (0.53%)	0.00	2014	SHEEDY FJ	132	0.00

### Journals and cocited academic journals

Visualization analysis of journals was performed by VOSviewer. The results showed that 894 articles on “inflammasome AND atherosclerosis” were published in 626 academic journals. *The journal International Journal Of Molecular Sciences* (25, 2.67%) produced the most studies, followed by *Arteriosclerosis Thrombosis And Vascular Biology* (22, 2.35%). As shown in Table 4, *Circulation* (29.69) is the leader in impact factor (IF). Moreover, Table 4 illustrates that 20% of journals are from Q1. According to Table 4, *Nature* (751) and *Circulation* (629) have the first and second highest citations, respectively. Journal Citation Reports (JCR) for 2021 show that half of the top 10 cocited journals were located in the Q1 region.

**Table 4:** Top 10 journals and cocited journals related to “inflammasome AND atherosclerosis”.

Rank	Journal	Count (%)	IF(2021)	JCR	Cocited journal	Citation	IF(2021)	JCR
1	International Journal Of Molecular Sciences	25 (2.67%)	6.208	Q2	Nature	751	69.504	Q1
2	Arteriosclerosis Thrombosis And Vascular Biology	22 (2.35%)	10.514	Q2	Circulation	629	39.918	Q1
3	Circulation Research	21 (2.24%)	23.213	Q1	Arteriosclerosis Thrombosis And Vascular Biology	620	1.514	Q2
4	Frontiers In Pharmacology	21 (2.24%)	5.988	Q2	Plos One	596	3.752	Q2
5	Atherosclerosis	20 (2.13%)	6.847	Q2	Circulation Research	571	23.213	Q1
6	Circulation	19 (2.02%)	39.918	Q1	Journal Of Biological Chemistry	535	5.486	Q2
7	Frontiers In Immunology	19 (2.02%)	8.786	Q2	Nature Immunology	531	31.25	Q1
8	Biochemical And Biophysical Research Communications	18 (1.92%)	3.322	Q2	European Heart Journal	522	35.855	Q1
9	Scientific Reports	15 (1.60%)	4.996	Q2	Journal Of Immunology	506	5.426	Q2
10	Plos One	14 (1.49%)	3.752	Q3	Atherosclerosis	505	6.847	Q2

**Figure 5:**
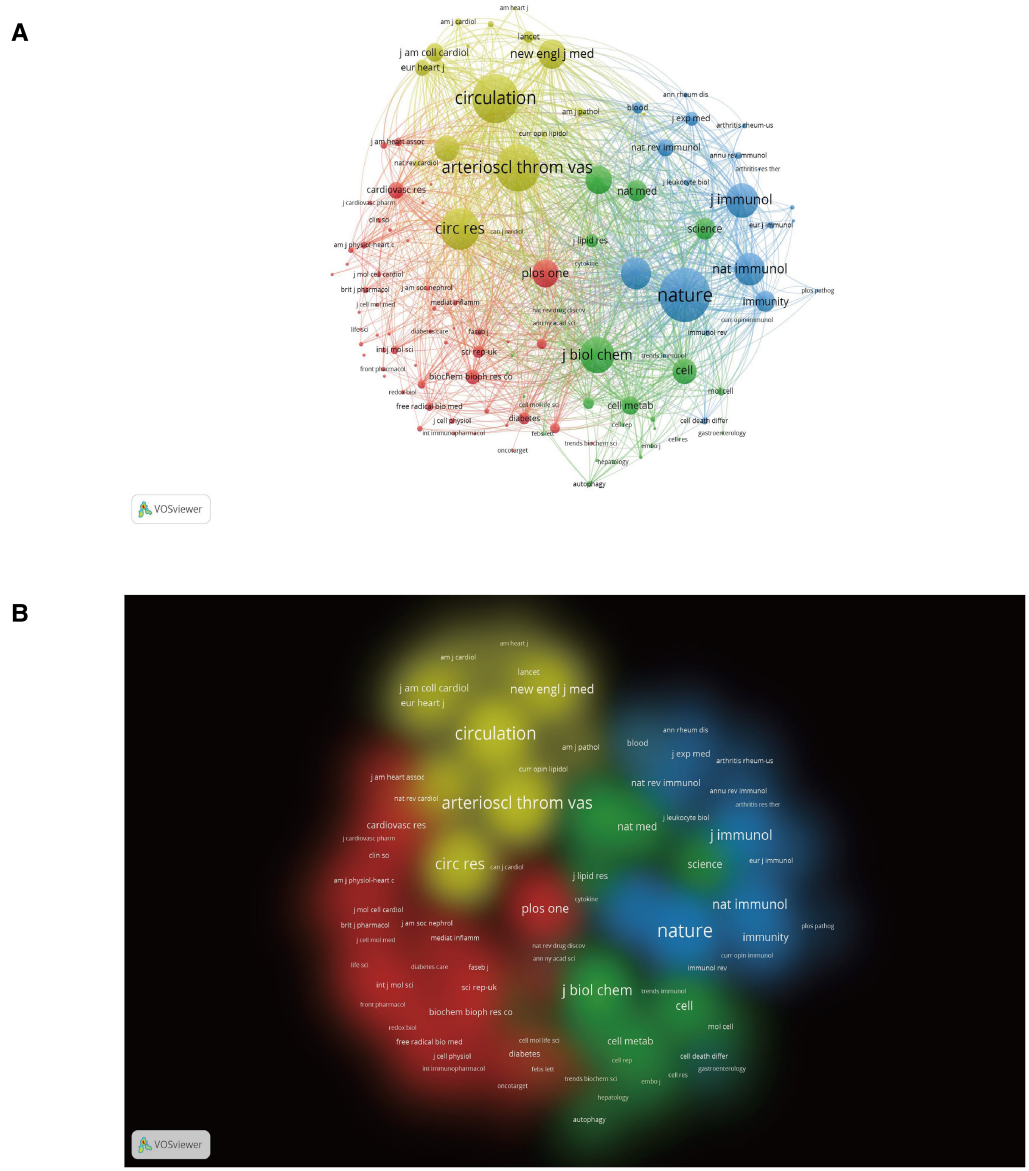
Analysis of cocited journals based on the VOSviewer visual map. (**A**) Network visualization; (**B**) Density visualization. Nodes on the map are colored according to their element density.

Figure 5 shows which journals have had a leading role and a profound impact on the development of scholarship in the field. Each node's size and density are proportional to the number of journal citations it represents. However, there is a positive relationship between the width of each line and the strength of the connection between two nodes. Four clusters can be enriched from the results of the cocited journals analysis, as shown in Figure 5. The largest cluster (yellow) includes journals focused on cardiovascular clinical and basic research. The more prominent journals in this cluster are *Circulation*, *Circulation Research*, *Arteriosclerosis Thrombosis and Vascular Biology*, and *European Heart Journal*.

### Keywords

Keywords are at the heart of every paper (Table 5). By analyzing the keywords, we summarize the hot topics in a specific field and identify future research directions. NLRP3, NF-kappaB, NLRP3 macrophage, activation, expression and oxidative stress appeared most frequently in this study, except for atherosclerosis (268).

**Table 5:** Top 20 keywords related to “inflammasome AND atherosclerosis”.

Rank	Keywords	Count	Centrality	Rank	Keywords	Count	Centrality
1	nlrp3 inflammasome	319	0.03	11	Cell	82	0.02
2	Atherosclerosis	268	0.02	12	nalp3 inflammasome	80	0.03
3	Activation	217	0.18	13	Smooth muscle cell	79	0.01
4	Expression	136	0	14	Low density lipoprotein	71	0.09
5	Oxidative stress	122	0.04	15	c reactive protein	69	0.01
6	Cardiovascular disease	121	0.01	16	Endothelial cell	64	0.07
7	Disease	105	0.05	17	Myocardial infarction	61	0
8	Mechanism	101	0.03	18	Apoptosis	54	0.14
9	nf kappa b	91	0.08	19	Mice	52	0.03
10	Macrophage	83	0.01	20	Rceptor	51	0.02

**Figure 6:**
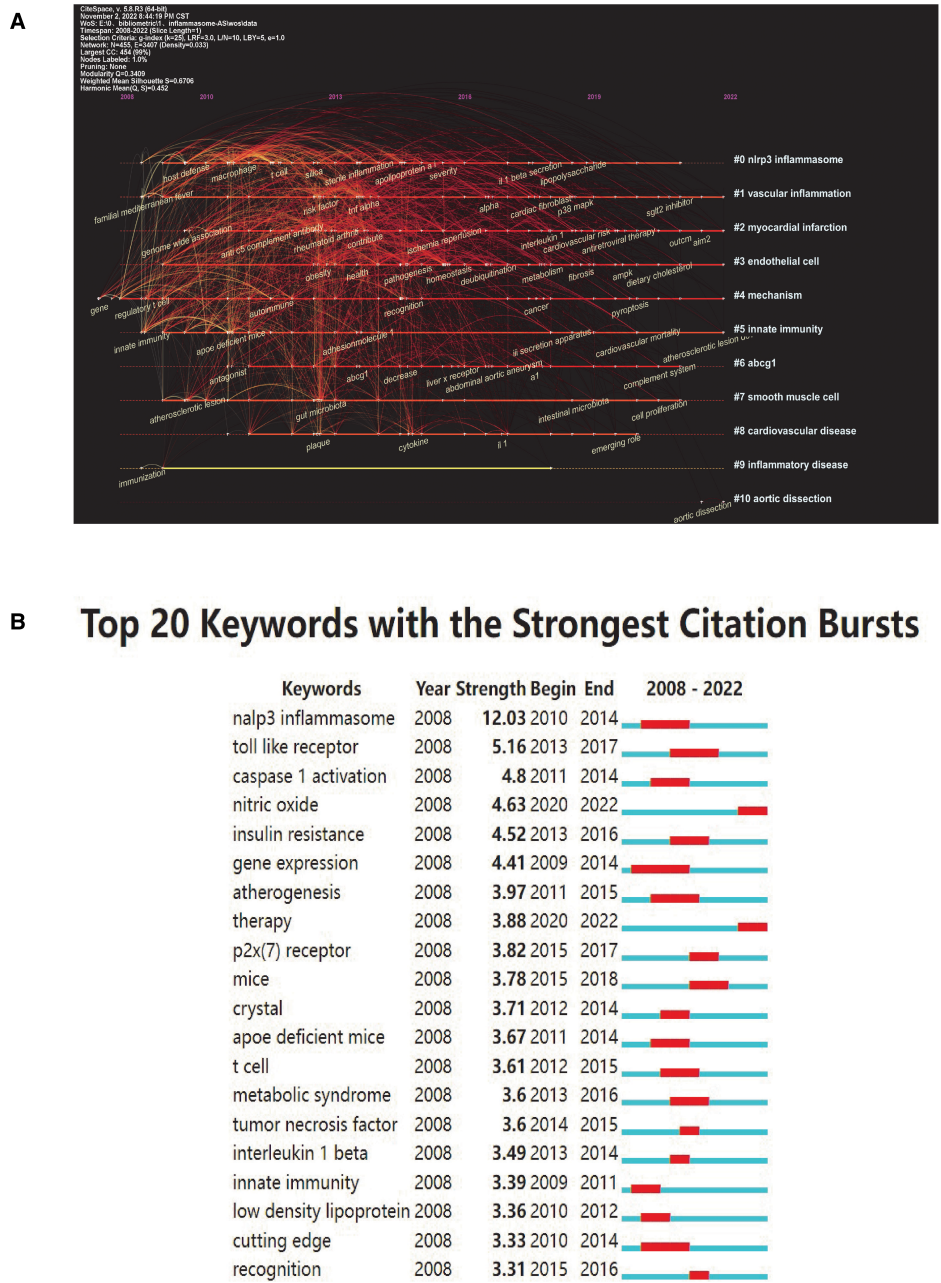
Keyword mapping. (**A**) Timeline distribution of keyword cluster analysis. Each horizontal line represents a cluster; the smaller the number, the larger the cluster, and #0 is the largest. The time is at the top, and keywords are located at their first co-occurrence time in the cluster. Cluster labels were extracted from title and abstract information by LLR. LLR, log-likelihood ratio. (**B**) Top 20 keywords with the strongest citation bursts (sorted by strength). The red bars indicate citation burstness.

To distill domain research themes more effectively and intuitively, we use Citespace software's unique timeline view. The timeline view (Figure 6A) presented high-frequency keywords in each cluster over time. We can see that five of the ten clusters are still ongoing. Among them, #1 (vascular inflammation) was the largest cluster, followed by #2 (myocardial infarction), #3 (endothelial cell), #4 (mechanism) and #5 (innate immunity). Cluster #0 (nlrp3 inflammasome) has the strongest aggregation and attention in the whole study of inflammasomes and AS, which indicates that studying their interactions is favored by researchers in this field.

Keyword bursts are those that were cited significantly frequently over a period. As shown in Figure 6B, the nlrp3 inflammasome had the strongest bursts (strength = 12.03), followed by Toll-like receptor (strength = 5.16) and caspase 1 activation (strength = 4.8). Notably, nitric oxide and therapy were in burstness until 2022.

### Cocited references and references burst

**Figure 7:**
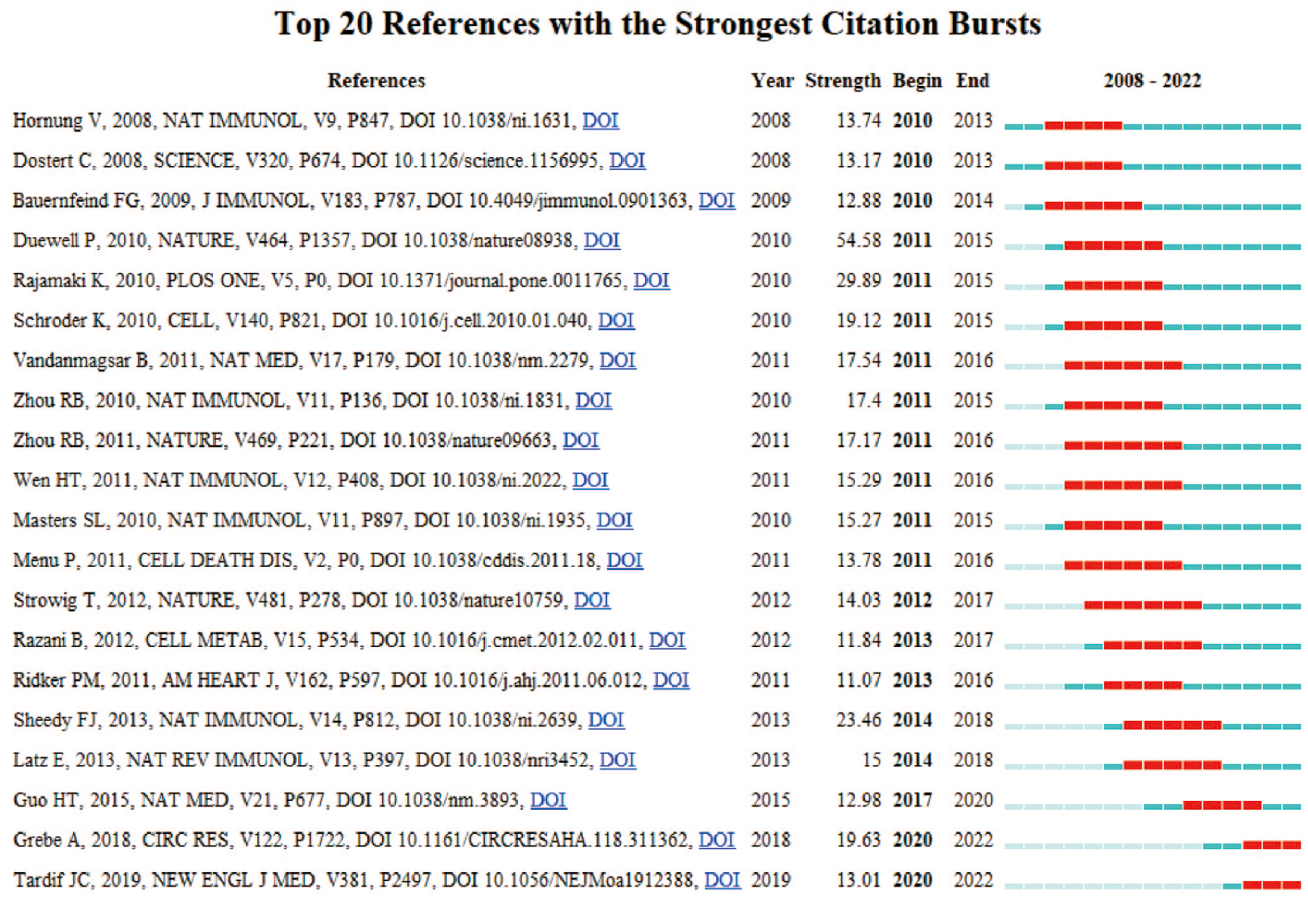
Top 20 references with the strongest citation bursts by CiteSpace.

In research, cocitation indicates that more than one article is cited by more than one work simultaneously and the two articles constitute a cocitation relationship. We retrieved 866 cocited references, and Table 6 illustrates the top fifteen most frequently cited references, of which *Antiinflammatory Therapy with Canakinumab for Atherosclerotic Disease* is the most frequently cited (211). Based on the strongest burst of cocited references, Figure 7 illustrates that the first burst occurred in 2010.

**Table 6:** Top 15 cocitation of cited reference on “inflammasome AND atherosclerosis”.

Rank	Reference	Citation	Year	Centrality
1	Antiinflammatory Therapy with Canakinumab for Atherosclerotic Disease	211	2017	0.03
2	NLRP3 inflammasomes are required for atherogenesis and activated by cholesterol crystals	128	2010	0.01
3	NLRP3 Inflammasome and the IL-1 Pathway in Atherosclerosis	113	2018	0.01
4	CD36 coordinates NLRP3 inflammasome activation by facilitating intracellular nucleation of soluble ligands into particulate ligands in sterile inflammation	70	2013	0.07
5	Cholesterol crystals activate the NLRP3 inflammasome in human macrophages: a novel link between cholesterol metabolism and inflammation	70	2010	0.01
6	Inflammasomes: mechanism of action, role in disease, and therapeutics	69	2015	0.05
7	NLRP3 inflammasome: Its regulation and involvement in atherosclerosis	68	2018	0.01
8	NLRP3 Inflammasome Inhibition by MCC950 Reduces Atherosclerotic Lesion Development in Apolipoprotein E-Deficient Mice-Brief Report	63	2017	0.06
9	Role of NLRP3 Inflammasomes in Atherosclerosis	57	2017	0.03
10	Mechanism and Regulation of NLRP3 Inflammasome Activation	56	2016	0.02
11	Interleukin-1 and the Inflammasome as Therapeutic Targets in Cardiovascular Disease	18	2020	0.01
12	VX-765 attenuates atherosclerosis in ApoE deficient mice by modulating VSMCs pyroptosis	10	2020	0.01
13	Macrophages in Atherosclerosis Regression	9	2020	0.01
14	The AIM2 inflammasome exacerbates atherosclerosis in clonal haematopoiesis	5	2021	0.01
15	Specific NLRP3 Inhibition Protects Against Diabetes-Associated Atherosclerosis	5	2021	0.01

## Discussion

### General information

Over 20 years have passed since the inflammasome was proposed to be involved in cardiovascular disease. This work involves annual publications increasing steadily over a 20-year period, which will continue in 2022. Our analysis of the burst of keywords suggests that more researchers are looking into inflammasomes, which remain a hot research topic, and there will be an increase in literature related to inflammasomes in the coming decade. Inflammasome dominants working on atherosclerosis are most commonly found in Western countries, organizations, authors, and journals.

### Hot spots and trending

In complex networks, node centrality represents the importance of a node. It is largely used to gauge a node's contribution to the overall network structure by serving as a bridge. Table 2 shows that the USA tops the list in centrality (0.53), meaning it plays an essential role in international cooperation. In addition, four of the top ten research institutions are from China, while three are from the US, with Harvard Medical School (0.11) having the most impact. Nevertheless, as shown in Figure 3, a dispersed distribution of nations and institutions can be observed. However, some prestigious research institutions with a high academic reputation, such as Shanghai Jiao Tong University and University of Massachusetts, have not formed their own unique network of academic communication and interaction, which proves that many prestigious universities are closed in the field of inflammasome research, which is not conducive to the exchange of academic results and multidisciplinary field cooperation. Therefore, it is highly recommended for China, the United States, and other countries to remove academic barriers to promote cooperation and communication among their research institutions.

According to document analysis, the Eicke Latz team, Yong Zhang team, and Peter Libby team still lead this field. The Eicke Latz team focused on cholesterol crystals in atherosclerotic lipid plaques activating inflammasomes to induce the secretion of proinflammatory interleukin-1 family cytokines and exacerbating the pathological process of atherosclerosis (43, 48–50). Duewell et al. observed that minute cholesterol crystals are found in early diet-induced atherosclerotic lesions of ApoE^−/−^ mice, and these crystalline substances can cause inflammation by stimulating the caspase-1-activating NLRP3 inflammasome, which plays an important role in cleavage of IL-1 family cytokines. In addition, the results showed that preconditioning mice deficient in low-density lipoprotein receptor (LDLR) transplantation with bone marrow-induced NLRP3-deficient, ASC-deficient or IL-1*α*/*β*-deficient cells confers cardioprotection when they are fed a high-cholesterol diet. These findings indicated that crystalline cholesterol is involved in activation of the NLRP3 inflammasome during early atherosclerosis (43). Niyonzima et al. demonstrated that activation of the NLRP3 inflammasome in the vessel wall triggers plaque inflammation when cholesterol precipitates into cholesterol crystals (CCs), thus promoting the progression of atherosclerosis. They also found that the accumulation of complement C1q and the C5b-9 complex around CC-clefts and the high expression of complement receptors C5aR1, C5aR2 and C3aR1 in carotid plaques from patients with stable angina pectoris or ACS play a crucial role in the upregulation of NLRP3 inflammasome components (48).

Yong Zhang and his collaborators have many novel findings in their pharmacological research (51–54). Zhang et al. demonstrated that the cardioprotective effects of melatonin reduce atherosclerotic plaques in high-fat diet (HFD)-treated ApoE^−/−^ mice and induce the downregulation of NLRP3, cleaved caspase1, NF-kappa B/GSDMD, IL-1 beta, and IL-18 in the aortic endothelium. They also found that melatonin confers consistent antipyroptotic effects against ox-LDL in the human endothelium by decreasing lncRNA MEG3, which inhibits miR-223 and activates NLRP3, thus enhancing pyroptosis. These results indicated that melatonin-induced cardiac protection is associated with the MEG3/miR-223/NLRP3 axis (51). Wu et al. demonstrated that nicotine expanded atherosclerotic plaque size while activating the inflammatory cytokine storm in ApoE^−/−^ mice. They also revealed that when preconditoning human aortic endothelial cells (HAECs) with nicotine, the activation of the NLRP3 inflammasome is closely associated with cleavage of caspase-1, resulting in secretion of IL-1β and IL-18, which are abolished by a caspase-1 inhibitor. Furthermore, reactive oxygen species (ROS) may play a crucial role in the nicotine-NLRP3-ASC-pyroptosis pathway because endothelial cell pyroptosis is inhibited by an ROS scavenger (N-acetyl-cysteine, NAC) (52). Zhang et al. found that matrine confers cardioprotection against advanced glycation end product (AGE)-induced HAEC injury by reducing ROS and decreasing NLRP3, ASC, caspase-1, p20 and IL-1β expression, which subsequently recovered HAEC viability. Furthermore, ROS, NLRP3, caspase-1, and IL-1β form a negative loop in AGE-induced HAECs, explaining the quantitative threshold of matrine to perform cardioprotection (53).

Peter Libby and his collaborators focused on the potential value of using IL-1β as a treatment target for atherosclerosis, including performing biomarker-directed application of an anti-inflammatory strategy and personalized allocation of therapy for cardiovascular patients (55, 56). Ridker et al. illustrated that canakinumab confers a cardioprotective effect against atherosclerosis in patients with incident lung cancer by reducing the NLRP3 inflammasome, which subsequently inhibits the downstream signaling pathway of IL-1β. Interestingly, compared to the placebo group, canakinumab significantly lowered total cancer mortality (*p *= 0.0007 for trend across groups) in patients with atherosclerosis who had myocardial infarction during a median follow-up of 3.7 years (55). In another clinical study, Ridker et al. demonstrated that stable postmyocardial infarction patients with chronic kidney disease treated with canakinumab had a lower incidence of major adverse cardiovascular events than placebo patients. The reason is that as a human monoclonal antibody targeting IL-1β, canakinumab might exert cardiovascular benefits by mediating the activation of IL-1β and the NLRP3 inflammasome within the kidney, thus contributing to cardioprotection during atherosclerosis (56). While many emerging researchers are studying inflammasomes in atherosclerosis, the literature analysis suggests that there is a lack of communication and collaboration with international teams at the moment. Research in this field needs to be promoted by more exchanges and collaborations in the future.

In the last two decades, inflammation research on atherosclerosis has flourished. Keyword analysis in this research shows that different cardiovascular cells (including macrophages, smooth muscle cells, and endothelial cells) are injured when they endure redox regulation, mitochondrial dysfunction, and calcium overload by stimulating inflammasomes (4, 9, 57). Macrophages play an essential role in integrating inflammatory and metabolic signals in plaque formation (58). Macrophages are recruited by messengers to migrate across endothelial cells and subsequently phagocytose lipids, initiating innate immune responses and inflammation by boosting the combination of Toll-like receptors with their ligands and activating the NLRP3 inflammasome (58–60). Cholesterol crystals are present in the extracellular space and within macrophages in atherosclerotic plaques. Currently, confocal reflectance microscopy is performed to explore whether cholesterol crystals can also be found in early lesions in ApoE^−/−^ mice, even though this feature has previously been considered a feature of advanced plaques (61). The results proved that cholesterol crystals phagocytosed by macrophages cause NLRP3 inflammasome activation. Sheedy et al. reported that when macrophages phagocytose cholesterol crystals, they also activate their own intracellular cholesterol synthesis and activate NLRP3 (62). CD36 was found to play a key role in the production and nucleation of cholesterol crystals in macrophages treated with OX-LDL and subsequent lysosomal destruction and NLRP3 inflammasome activation. Interestingly, when CD36 was inhibited, macrophages could not respond to OX-LDL-induced lipid peroxidation signals, and targeted knockdown of CD36 in mice attenuated inflammation, as well as cholesterol crystals in atherosclerotic plaques (62). Notably, CD36 can be involved in pathological processes associated with Alzheimer's disease and type 2 diabetes by mediating the uptake of amyloid-forming peptides and thereby activating NLRP3, suggesting signaling crosstalk between lysosome-mediated NLRP3 activation and the reaggregation and conversion of soluble ligands (63).

**Figure 8:**
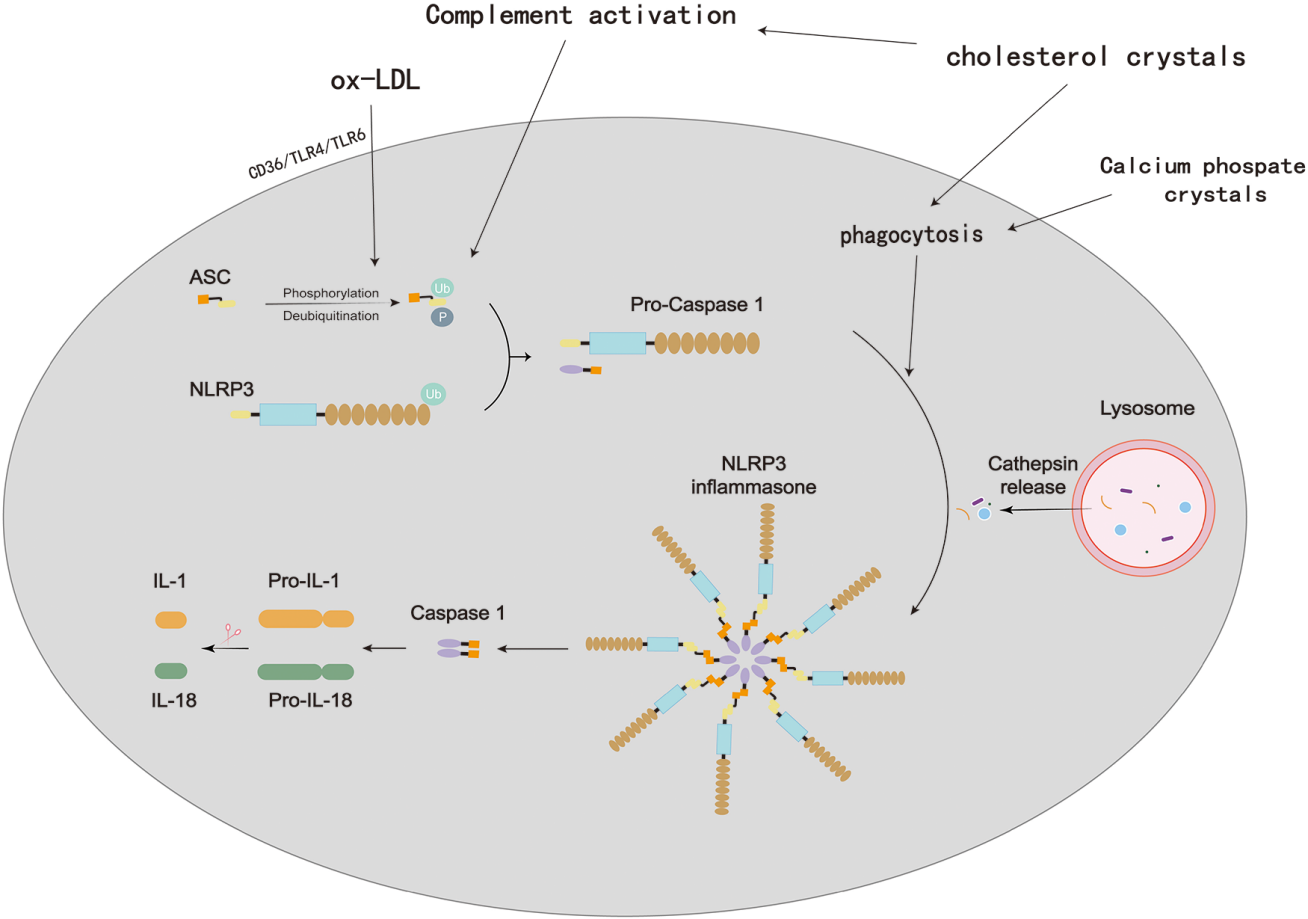
Process of NLRP3 inflammasome activation in atherosclerosis. In atherosclerotic lesions, oxidized low-density lipoprotein (oxLDL) and cholesterol crystals (CCs) are prevalent and promote a self-sustaining cycle of continuous NLRP3 inflammasome priming and activation in macrophages. OxLDL binds to CD (cluster of differentiation)36, which triggers oxLDL phagocytosis and a priming signal *via* TLR (Toll-like receptor)4/TLR6. Lysosomal damage brought on by the phagocytosis of extracellular CCs results in the activation of the NLRP3 inflammasome. Additionally, CCs stimulate the complement system, which encourages CC phagocytosis and consequently, NLRP3 activation.

Although the mechanism remains unknown, other crystalline or amyloid-like substances in atherosclerotic plaques, such as calcium phosphate crystals or serum amyloid A, may also represent DAMPs, which can trigger inflammasome activation and IL-1β secretion (Figure 8) (63). Xiao et al. proved that NLRP3 activation, provoked by atherosclerosis-prone blood flow, is important for the progression of atherosclerosis (64). By overexpressing inflammation-related receptors in VSMCs, arterial blood flow disturbances have been found to induce phenotypic changes, and these changes are highly correlated with inflammatory responses (64). Wen et al. demonstrated that beta-glycerophosphate induces vascular smooth muscle cells (VSMCs) to upregulate the expression of mRNA levels of the Nalp3 inflammasome (an old name for NLRP3 inflammasome), including Nalp3, ASC and caspase1, thus resulting in extensive calcification in cultured VSMCs (65). They also found that after NALP3-siRNA transfection, VSMC calcification was attenuated, and further study of clinical popliteal artery specimens suggested that compared with their noncalcified adjacent tissues, inflammasome complex mRNA levels and caspase1 were upregulated in calcified tissues. These data indicated the crucial roles of the Nalp3 inflammasome in VSMC calcification, which is closely correlated with increased morbidity and mortality in patients with atherosclerosis (65). With further research, scholars have revealed that VSMCs transition into inflammatory phenotypes when exposed to OxLDL, which also contributes to local inflammation (66, 67). The oxidized phospholipids in low-density lipoprotein, including oxPAPC, may induce multiple proatherogenic events in macrophages and endothelium, macrophages, and VSMCs (65–67). Byon et al. explored the effect of Txnip on VSMC inflammation by treating VSMCs from both Txnip^−/−^ and ApoE^−/−^ mice with OxPAPC or H_2_O_2_ in DMEM containing 1% FBS for 4 h (68). The results showed that ablation of Txnip exerts cardioprotection by reducing the inflammatory cytokines and adhesion molecules in VSMCs, thus attenuating cellular oxidative stress when threatened by oxidized phospholipids and hydrogen peroxide (68).

Diabetes, hypertension and coronary artery disease are all associated with endothelial dysfunction, a marker and predictor of adverse cardiovascular events (69). Chronic inflammation, tissue swelling, and thrombosis may result from deteriorated endothelial dysfunction (70). Previous research has illustrated that vascular endothelial cells at sites of inflammation are closely associated with the regulation of inflammatory progression and are crucial for cardiovascular homeostasis as dynamic adaptive interfaces (71–73). Boini et al. demonstrated that the colocalization of NLRP3 with ASC or NLRP3 with caspase-1 and IL-1β production is upregulated in mouse carotid arterial endothelial cells after treatment with trimethylamine-N-oxide (TMAO) overnight, which is abolished by a caspase-1 inhibitor during endothelial injury (74). Furthermore, they indicated that both redox regulation and lysosomal dysfunction play crucial roles in TMAO-induced activation of NLRP3 inflammasomes (74). In addition, Li et al. demonstrated that miR-30c-5p confers a cardioprotective effect on HAECs from ox-LDL-induced pyroptosis, which is closely associated with the NLRP3 inflammasome (75). The results suggested that anti-miR-30c-5p-induced activation of the NLRP3 inflammasome and pyroptosis are abolished by silencing forkhead Box O3 (FOXO3) in HAECs, indicating that FOXO3 plays a crucial role in NLRP3-mediated pyroptosis (75).

### The crosstalk of inflammasomes with other cardiovascular diseases

Inflammasomes are implicated in atherosclerosis as well as other cardiovascular diseases, such as heart failure, atrial fibrillation (AF) and cardiomyopathy (76–78). The mechanisms and biological functions of inflammasomes involved in other highly prevalent cardiovascular diseases are important references for our study of their role in atherosclerosis. The inflammasome plays an important role in the development of heart failure. Sano et al. explored the effects of the NLRP3 inflammasome on heart failure by using both Tet2^−/−^ and Tet2^+/+^ mice to undergo permanent ligation of the left anterior descending artery, followed by transverse aortic constriction (79). The results showed that Tet2 mutations within hematopoietic cells exaggerate heart failure in Tet2-deficient but not in wild-type mice. The reason is that Tet2 deficiency triggers NLRP3 inflammasome-mediated IL-1β signaling, thus contributing to worsened cardiac remodeling and function, which is abolished by MCC950 (a selective NLRP3 inhibitor) in mouse heart (79). Mezzaroma et al. observed that the formation of the NLRP3 inflammasome results in worsened loss of functional myocardium in the mouse heart after AMI, thus contributing to heart failure (80). Furthermore, NLRP3 inflammasome-induced cardiomyocyte death and cardiac remodeling after AMI are aborgated by inhibiting P2X7, which plays an important role in inflammasome formation and caspase-1 activation during AMI, indicating that the NLRP3 inflammasome is a potential target for the prevention of heart failure (80).

There has also been evidence that the NLRP3 inflammasome is linked to atrial fibrillation progression. Yao et al. found that the NLRP3 inflammasome is activated in the atrial cardiomyocytes of patients with paroxysmal AF and chronic AF. They found that NLRP3 overexpression in mice induced spontaneous premature atrial contractions, and this inducible AF was abolished by an NLRP3 inflammasome inhibitor (MCC950) (81). Moreover, the results showed that preconditioning cardiomyocyte-specific knockdown of NLRP3 in mice confers protection against AF development, indicating that inhibition of NLRP3 could be a novel therapeutic strategy in AF (81). One recent study showed that the expression of activated components of NLRP3 is increased in atrial whole-tissue homogenates and cardiomyocytes from patients with postoperative atrial fibrillation (POAF) (81). Heijman et al. demonstrated that preconditioning a mouse atrial cardiomyocyte cell line (HL-1-cardiomyocytes) with IL-1β induced NLRP3 signaling activation, as well as spontaneous sarcoplasmic-reticulum (SR) Ca^2+^-release events (82). They also revealed that some molecular substrates, such as Ca^2+^/calmodulin-dependent protein kinase-II (CaMKII) and ryanodine-receptor channel type-2 (RyR2), are significantly activated in POAF-cardiomyocytes and HL-1-cardiomyocytes, which constitute the NLRP3-inflammasome/CaMKII signaling pathway, contributing to the deterioration of POAF (82).

The NLRP3 inflammasome is a crucial cytokine that promotes the development of cardiomyopathy. Ye et al. demonstrated that dapagliflozin, a sodium-glucose cotransporter 2 inhibitor, exerts cardioprotection in type 2 diabetic mice by attenuating the protein levels of NALP3, ASC, IL-1β, IL-6, and caspase-1 in mouse hearts, thereby inhibiting the activation of the NLRP3 inflammasome (83). Using saxagliptin, a DDP4 inhibitor, they further found that the protective effect of dapagliflozin is augmented, and dapagliflozin attenuates fibrosis and deterioration of the left ventricular ejection fraction in type 2 diabetic mice by downregulating the P-AMPK/total-AMPK ratio. These data indicated that diabetic cardiomyopathy remodeling can be prevented by suppressing the NLRP3 inflammasome (83). Li et al. found that sepsis and sepsis-induced cardiomyopathy are closely associated with inflammation, cardiomyocyte apoptosis and pyroptosis, and further study suggested that stimulator of interferon genes (STING) promotes phosphorylated interferon regulatory Factor 3 (IRF3) translocation into the nucleus and subsequently increases the expression of NLRP3 in wild-type mice during LPS stimulation (84). They also revealed that STING^−/−^ mice exhibit improved cardiac function, and NLRP3 exaggerates LPS-induced injury in wild-type but not in STING^−/−^ cardiomyocytes (84).

### Frontiers and prospects for inflammasome research

The relationship between inflammasomes and sterile inflammatory diseases, such as atherosclerosis, myocardial infarction, heart failure, atrial fibrillation and cardiomyopathy, has been further explored in previous studies. Smoking, drinking, sedentary lifestyle, high salt and sugar diet, and irregular lifestyle habits are common risk factors for cardiovascular disease. In the case of hyperstimulation by these pathologies, the transcriptional and protein assembly processes of the inflammasome are initiated in the cytoplasm, which consecutively leads to increased synthesis and release of a range of inflammatory factors, i.e., the onset of an inflammatory factor storm. However, it has been suggested that inflammasomes may be a double-edged sword in some cancers, depending on the cell and pathological condition in which they are synthesized. Inflammasome activation in acute disorders aids in the removal of dead cells and the beginning of tissue healing. However, chronic disease-related persistent inflammasome activation is harmful. At the same time, there is a serious lack of evidence from clinical trials on whether inflammasomes can be used as selective targets for cardiovascular disease therapy; therefore, signaling molecules that mediate the positive and negative effects of inflammasome activation need to be urgently explored. Furthermore, the mechanisms that regulate noncanonical inflammasome signaling and their communication with canonical inflammasomes may be crucial for exploring more successful therapeutic approaches for a wide variety of pathological circumstances.

## Limitations

A bibliometric analysis of inflammasomes in atherosclerosis is presented here for the first time; however, there are some limitations to recognize. First, we set the end date for the research search to March 29th, 2022, but at the same time, new studies were still being published in the WOS database, so this portion of the studies was not included in the final bibliometric pool. Second, due to methodological and software algorithm limitations, only articles and reviews were included in our research, and other types of studies involving inflammasomes were not summarized. Moreover, the quality of the publications included in the WOS database varies, and researchers focus on different research hotspots at different times, which may reduce the accuracy of the topological analysis. Finally, we encourage international scholarly exchange among researchers worldwide, but this study did not include studies published in languages other than English.

## Conclusion

We performed a bibliometric analysis of inflammasomes in atherosclerosis research knowledge maps, potential hotspots, and future trends. The United States and China are still powerful in inflammasome research because researchers in these two countries are highly productive, have strong scholarly impact and are highly involved in academic exchanges. The primary author who had a critical academic influence on the field was EICKE LATZ. The *International Journal of Molecular Sciences*, *Arteriosclerosis Thrombosis and Vascular Biology*, *Circulation Research*, *Frontiers in Pharmacology*, *Atherosclerosis*, *Circulation*, *Frontiers in Immunology*, *Biochemical and Biophysical Research Communications* were regarded as key journals in the present research. Moreover, we identified NLRP3, NF-kappaB, NLRP3 macrophages and oxidative stress as research topics in inflammasomes in atherosclerosis research.

## Data Availability

The raw data supporting the conclusions of this article will be made available by the authors, without undue reservation.
